# Supplementary material for: Content-rich biological network constructed by mining PubMed abstracts
Source: BMC Bioinformatics. 2004 Oct 8;5:147. doi: 10.1186/1471-2105-5-147 (PMC528731; doi:10.1186/1471-2105-5-147)
Supplement: Additional File 2 — The original results of the above study (non-essential files are deleted to keep the file size under the limit set by BMC bioinformatics). [file 1471-2105-5-147-S2.bz2 › chilibotAdditionalFile2/dip50/354ID1495999E4996/html/IL5_IL5RA.html]

 


 **IL5** and **IL5RA** 
  
Found 67 abstracts in PubMed, retrieved 50.  
 

 What does Google say? 
 PDF only 
| .edu only 

---

**Interactive relationship** (e.g. stimulation, inhibition, etc)

**Stimulatory relationship**- The high affinity receptor R for IL 5  [ **IL5** ]  consists of a unique alpha chain IL 5R alpha  [ **IL5RA** ]  and a beta chain beta c that is shared with the receptors for IL 3 and granulocyte macrophage colony stimulating factor GM CSF.  Ref: 8671609 Int Immunol, 1996
- Moreover, endogenous STAT3 was tyrosine phosphorylated and activated in human IL 5  [ **IL5** ]  stimulated BaF3 cells ectopically expressing the human IL 5R BaF3 IL5R  [ **IL5RA** ] .  Ref: 7592760 J Biol Chem, 1995
- The high affinity receptor for IL 5  [ **IL5** ]  IL 5R is composed of an alpha subunit IL 5R alpha  [ **IL5RA** ]  expressed by the eosinophil lineage, that associates with a beta c subunit shared with the receptors for IL 3 and granulocyte macrophage colony stimulating factor GM CSF.  Ref: 7836416 J Biol Chem, 1995
- The receptor for interleukin 5  [ **IL5** ]  IL 5R is composed of a unique alpha chain IL 5R alpha  [ **IL5RA** ]  expressed on eosinophils and basophils, associated with a beta c subunit, which is shared by the receptors for IL 3 and granulocyte macrophage colony stimulating factor.  Ref: 7592760 J Biol Chem, 1995
- Interleukin 5  [ **IL5** ]  receptor alpha subunit gene regulation in human eosinophil development identification of a unique cis element that acts lie an enhancer in regulating activity of the IL 5R alpha  [ **IL5RA** ]  promoter.  Ref: 8585949 Curr Top Microbiol Immunol, 1996
**Neutral relationship**- Detailed analysis of the IL 5  [ **IL5** ]  IL 5R alpha  [ **IL5RA** ]  interaction characterization of crucial residues on the ligand and the receptor.  Ref: 7628440 EMBO J, 1995
- These results strongly suggest that the lineage specificity of IL 5  [ **IL5** ]  is mainly due to the restricted expression of IL 5R alpha  [ **IL5RA** ] .  Ref: 7869049 J Exp Med, 1995
- These results support the notion that the lineage specificity of IL 5  [ **IL5** ]  is mainly due to the restricted expression of IL 5R alpha  [ **IL5RA** ] .  Ref: 8906104 Int Arch Allergy Immunol, 1996
- Using this surface, we quantitated for the first time direct binding of s beta c to IL5R  [ **IL5RA** ]  alpha complexed to either wild type or single chain  **IL5** .  Ref: 12202242 Anal Biochem, 2002
- More detailed characterization reveals that the down regulation of IL 5R alpha  [ **IL5RA** ]  mRNA is specific to IL 3, IL 5  [ **IL5** ] , and GM CSF.  Ref: 9574547 J Immunol, 1998
- The membrane proximal proline rich sequence of the cytoplasmic domain of IL 5R alpha  [ **IL5RA** ]  was found to be essential for the IL 5  [ **IL5** ]  induced proliferative response, expression of nuclear proto oncogenes such as c jun, c fos and c myc, and activation of Bruton s tyrosine and JAK2 kinases.  Ref: 7473055 Yakugaku Zasshi, 1995
- Biosensor analysis of dynamics of interleukin 5  [ **IL5** ]  receptor subunit beta c interaction with  **IL5**  IL5R  [ **IL5RA** ]  alpha complexes.  Ref: 12202242 Anal Biochem, 2002
- In addition, transfectants expressing chimeric receptors which consist of the extracellular domain of IL 5R alpha  [ **IL5RA** ]  and the cytoplasmic domain of beta c responded to IL 5  [ **IL5** ]  for proliferation and tyrosine phosphorylation of JAK1.  Ref: 8671609 Int Immunol, 1996
- We therefore suggest that an IL 5  [ **IL5** ]  driven inflammation generates an eosinophil tissue phenotype that is characterized by a low IL 5R alpha  [ **IL5RA** ]  expression.  Ref: 12519389 Clin Exp Immunol, 2003
- By Northern blot analysis of mRNAs obtained from normal human blood eosinophils, we show in this report that the hematopoietic cytokines IL 5  [ **IL5** ] , IL 3, and GM CSF down regulate IL 5R alpha  [ **IL5RA** ]  mRNA while up regulating alpha chain mRNAs for both IL 3R and GM CSFR as well as the beta chain mRNA.  Ref: 9574547 J Immunol, 1998
- The IL 5R alpha  [ **IL5RA** ]  was down regulated, in a dose dependent manner, by recombinant IL 5  [ **IL5** ]  and GM CSF, with IL 5  [ **IL5** ]  being most potent.  Ref: 12519389 Clin Exp Immunol, 2003
- Experiments with neutralizing antibodies against IL 5  [ **IL5** ]  and GM CSF, revealed that IL 5  [ **IL5** ]  was the principal cytokine responsible for the down regulation of the IL 5R alpha  [ **IL5RA** ] .  Ref: 12519389 Clin Exp Immunol, 2003
- High affinity receptor for IL 5  [ **IL5** ]  IL 5R, a predominant eosinophil maturation factor, is composed of an IL 5  [ **IL5** ]  binding alpha chain IL 5R alpha  [ **IL5RA** ]  and a signal transducing beta chain that is shared by IL 3 and granulocyte macrophage CSF GM CSF receptors IL 3R and GM CSFR.  Ref: 9574547 J Immunol, 1998
- In conclusion our data indicate that IL 5  [ **IL5** ] , as opposed to its proposed action on eosinophil progenitors, down regulates the IL 5R alpha  [ **IL5RA** ]  chain on mature eosinophils.  Ref: 12519389 Clin Exp Immunol, 2003
- These aspects of IL 5  [ **IL5** ]  action on IL 5R alpha  [ **IL5RA** ]  expression could gain new insights into the mechanisms of specific immuno modulatory therapies, such as anti IL 5  [ **IL5** ] .  Ref: 12519389 Clin Exp Immunol, 2003
- The human interleukin 5  [ **IL5** ]  IL 5  [ **IL5** ]  receptor consists of an alpha chain that specifically binds the ligand with intermediate affinity, and a beta c chain, that associates with the IL 5  [ **IL5** ]  IL 5R alpha  [ **IL5RA** ]  complex, leading to a high affinity, signal transducing receptor complex.  Ref: 10092887 Eur J Biochem, 1999
- In contrast to the mAbs, none of the Fabs blocked binding of 125I IL 5  [ **IL5** ]  to IL 5R alpha  [ **IL5RA** ]  or neutralized the biologic activity of IL 5  [ **IL5** ] .  Ref: 7759873 J Immunol, 1995

**Non-interactive relationship** (e.g. studied together, co-existance, homology, etc.)

- Incubation of eosinophils with IL 3, IL 5  [ **IL5** ] , or GM CSF led to reduced expression of IL 5R alpha  [ **IL5RA** ] , which was sustained for up to 5 days.  Ref: 12759409 J Immunol, 2003
- The IL 5  [ **IL5** ]  receptor alpha IL 5R alpha  [ **IL5RA** ]  is required for ligand specific binding while association with the beta chain results in increased binding affinity.  Ref: 7628440 EMBO J, 1995
- We infected interleukin 5  [ **IL5** ]  IL 5  [ **IL5** ]  transgenic IL 5  [ **IL5** ]  Tg and IL 5  [ **IL5** ]  receptor alpha knockout IL 5R alpha  [ **IL5RA** ]  mice with Angiostrongylus cantonensis to determine the possible roles of IL 5  [ **IL5** ]  and eosinophils in A. cantonensis infection in mice.  Ref: 9211511 Parasitol Res, 1997
- Since message for the IL 5R alpha  [ **IL5RA** ] , IL 5R beta, and soluble IL 5R alpha  [ **IL5RA** ]  chains was detected in freshly isolated B cells, we further investigated whether IL 5  [ **IL5** ]  responsiveness to MCat, but not SAC, was due to their differential regulation of IL 5R mRNA.  Ref: 8568239 J Immunol, 1996
- Confirming the critical role of the IL5R  [ **IL5RA** ]  alpha cell surface expression level, treatment with suboptimal doses of a neutralising anti IL 5R alpha  [ **IL5RA** ]  antibody results in reduced activity of the mutant but not of wild type IL 5  [ **IL5** ] .  Ref: 10092887 Eur J Biochem, 1999
- OBJECTIVE This study was conducted to investigate the expression of alpha  **IL5**  and alpha GM CSF receptors alpha IL5r  [ **IL5RA** ]  and alpha GM CSFr mRNA in the paranasal sinus mucosa of atopic and nonatopic subjects with chronic sinusitis compared to controls.  Ref: 8902691 J Otolaryngol, 1996
- The addition of IL 9 to CD34 cells cultured in IL 3 and IL 5  [ **IL5** ]  enhanced eosinophil development, and IL 9 alone induced the expression of IL 5R alpha  [ **IL5RA** ] .  Ref: 10979962 Blood, 2000
- We generated interleukin 5  [ **IL5** ]  receptor alpha chain IL 5R alpha  [ **IL5RA** ]  deficient IL 5R alpha  [ **IL5RA** ]  mice by gene targeting.  Ref: 8630733 Immunity, 1996
- These Abs were tested for the ability to block binding of iodinated rIL 5 to the alpha chain of the human IL 5  [ **IL5** ]  receptor IL 5R alpha  [ **IL5RA** ]  and to inhibit proliferation of IL 5  [ **IL5** ]  dependent cells.  Ref: 7759873 J Immunol, 1995
- RT PCR was used to evaluate IL 5  [ **IL5** ]  receptor alpha IL 5R alpha  [ **IL5RA** ]  expression in various tissues and to assess bronchus and saphenous vein eosinophils through use of CCR3 expression.  Ref: 11897983 J Allergy Clin Immunol, 2002
- To investigate whether the lineage specificity of IL 5  [ **IL5** ]  is due to restricted expression of the IL 5  [ **IL5** ]  receptor alpha subunit IL 5R alpha  [ **IL5RA** ] , we generated transgenic mice that express the mouse IL 5R alpha  [ **IL5RA** ]  constitutively by phosphoglycerate kinase promoter.  Ref: 7869049 J Exp Med, 1995
- The IL 5R alpha  [ **IL5RA** ]  mice produced basal levels of eosinophils, while their bone marrow cells failed to form eosinophilic colonies in response to IL 5  [ **IL5** ] .  Ref: 8630733 Immunity, 1996
- By using capillary electrophoresis, a restriction map of genomic lambda phage clone of human interleukin 5  [ **IL5** ]  receptor alpha chain IL5R  [ **IL5RA** ]  alpha gene was constructed.  Ref: 9105179 Anal Chem, 1997
- We studied the expression of the IL 5  [ **IL5** ]  receptor alpha subunit IL 5R alpha  [ **IL5RA** ]  on bone marrow derived cluster of differentiation molecule 34 positive CD34 progenitor cells in asthmatics to prove the ability of progenitor cells to respond to IL 5  [ **IL5** ]  more readily.  Ref: 10584413 Changgeng Yi Xue Za Zhi, 1999
- The aim was to study in vitro regulation of the IL 5  [ **IL5** ]  receptor alpha IL 5R alpha  [ **IL5RA** ]  on purified peripheral blood eosinophils from healthy subjects.  Ref: 12519389 Clin Exp Immunol, 2003
- OBJECTIVE To investigate the iufluence of dexamethasone DM on expression of interleukin 5  [ **IL5** ]  receptor alpha IL 5R alpha  [ **IL5RA** ] , interleukin 3 receptor alpha IL 3R alpha, granulocyte macrophage colony stimulating factor receptor alpha GM CSFR alpha and common beta receptor beta cR in bronchalveolar lavage fluid BALF eosinophils in guinea pig asthma model, and to study the mechanism of promotion effect of steroid on eosinophil apoptosis.  Ref: 11758170 Zhonghua Jie He He Hu Xi Za Zhi, 2001
- What is the primary function of eosinophils ? Regulatory or anti inflammatory or pro inflammatory ? Results with IL 5  [ **IL5** ]  knockout mice and with IL 5R alpha  [ **IL5RA** ]  knockout mice clearly show that 1 eosinophils play key roles as cellular mediators in asthma and allergy,.  Ref: 11191098 Allergy Asthma Proc< MedlineTA>Allergy Asthma Proc, 2001
- RFX and 3 regulate expression of other medically important gene products for example, interleukin 5  [ **IL5** ]  receptor alpha chain, IL 5R alpha  [ **IL5RA** ] .  Ref: 10706293 Nature, 2000
- As part of our effort to define molecular recognition networks that lead to signalling, we investigated the effect of JAB on both JAK2 phosphorylation and JAK2 interaction state that ensue upon IL 5  [ **IL5** ]  stimulation in recombinant 293T cells cotransfected 293T cells with IL 5R alpha  [ **IL5RA** ] , beta c and hJAK2 either with or without JAB.  Ref: 10975987 Cytokine, 2000
- A soluble form of the human IL 5R alpha  [ **IL5RA** ]  chain IL 5Ra that contains the extracellular IL 5  [ **IL5** ]  binding domain has been evaluated for its effect on IL 5  [ **IL5** ]  binding to and activation of human eosinophils and basophils.  Ref: 9378992 J Immunol, 1997
- The results obtained with IL 5  [ **IL5** ]  knockout mice and with IL 5R alpha  [ **IL5RA** ]  knockout mice can be used to guide and inform asthma and allergy research in humans.  Ref: 11191098 Allergy Asthma Proc< MedlineTA>Allergy Asthma Proc, 1997
- To elucidate whether the lineage specificity of IL 5  [ **IL5** ]  is due to restricted expression of the IL 5  [ **IL5** ]  receptor alpha chain IL 5R alpha  [ **IL5RA** ] , transfectants of BCL1 B20 were established that express IL 3 receptor alpha BCL1 3R or GM CSF receptor alpha BCL1 GMR.  Ref: 8906104 Int Arch Allergy Immunol, 1996
- Studies in vitro on the phenotype of developing eosinophils and basophils suggest that the early acquisition of IL 5R alpha  [ **IL5RA** ] , as well as the capacity to produce cytokines such as GM CSF and IL 5  [ **IL5** ] , are features of the differentiation process.  Ref: 9698913 Mem Inst Oswaldo Cruz, 1997
- We defined two regions of IL 5R alpha  [ **IL5RA** ]  for the IL 5  [ **IL5** ]  induced proliferative response, the expression of nuclear proto oncogenes, and the tyrosine phosphorylation of cellular proteins including beta c, SH2 SH3 containing proteins and JAK2 kinase.  Ref: 8671609 Int Immunol, 1996
- The transgenic mouse expressed IL 5R alpha  [ **IL5RA** ]  ubiquitously, and the bone marrow cells formed various types of colonies, including multi lineage colonies, in response to IL 5  [ **IL5** ] .  Ref: 7869049 J Exp Med, 1995
